# *N*′-[(*E*)-2,3-Dihy­droxy­benzyl­idene]-2-meth­oxy­benzohydrazide

**DOI:** 10.1107/S1600536812042390

**Published:** 2012-11-03

**Authors:** Muhammad Taha, M. Syukri. Baharudin, Nor Hadiani Ismail, Syed Adnan Ali Shah, Sammer Yousuf

**Affiliations:** a Atta-ur-Rahman Institute for Natural Product Discovery, Universiti Teknologi MARA (UiTM), Puncak Alam Campus, 42300 Bandar Puncak Alam, Selangor D. E. Malaysia; b Faculty of Applied Science, Universiti Teknologi MARA (UiTM), 40450 Shah Alam, Malaysia; c Faculty of Pharmacy, Universiti Teknologi MARA, Puncak Alam, 42300, Selangor, Malaysia; d H.E.J. Research Institute of Chemistry, International Center for Chemical and Biological Sciences, University of Karachi, Karachi 75270, Pakistan

## Abstract

The title compound, C_15_H_14_N_2_O_4_ adopts an *E* conformation about the azomethine double bond. Intra­molecular N—H⋯O and O—H⋯N hydrogen bonds generate *S*(6) rings and help to establish the molecular conformation. The dihedral angle between the benzene rings is 17.84 (10)°. In the crystal, mol­ecules are linked by O—H⋯O and C—H⋯O hydrogen bonds into a two-dimensional network with a herring-bone pattern arranged parallel to the *bc* plane.

## Related literature

For applications and the biological activity of Schiff bases, see: Panneerselvam *et al.* (2009 ▶); Khan *et al.* (2009 ▶); Jarahpour *et al.* (2007 ▶). For related structures, see: Baharudin *et al.* (2012 ▶); Taha *et al.* (2012 ▶); Promdet *et al.* (2011 ▶).
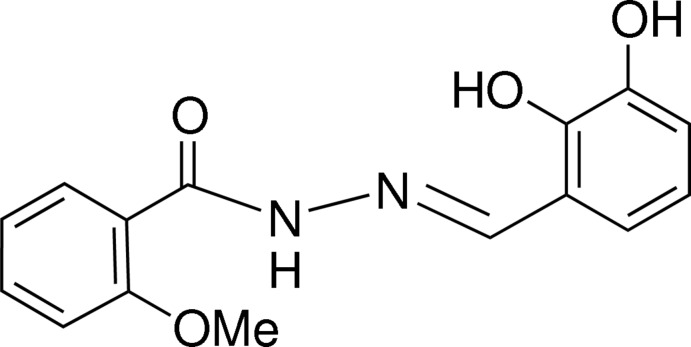


## Experimental

### 

#### Crystal data

C_15_H_14_N_2_O_4_
*M* *_r_* = 286.28
Orthorhombic, 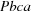

*a* = 14.1479 (17) Å
*b* = 8.6567 (11) Å
*c* = 22.570 (3) Å
*V* = 2764.2 (6) Å^3^
*Z* = 8
Mo *K*α radiation
μ = 0.10 mm^−1^
*T* = 273 K
0.56 × 0.18 × 0.04 mm

#### Data collection

Bruker SMART APEX CCD area-detector diffractometer
Absorption correction: multi-scan (*SADABS*; Bruker, 2000 ▶) *T*_min_ = 0.945, *T*_max_ = 0.996
15288 measured reflections
2574 independent reflections
1658 reflections with *I* > 2σ(*I*)
*R*_int_ = 0.042

#### Refinement

*R*[*F* ^2^ > 2σ(*F* ^2^)] = 0.042
*wR*(*F* ^2^) = 0.115
*S* = 1.03
2574 reflections
203 parameters
H atoms treated by a mixture of independent and constrained refinement
Δρ_max_ = 0.14 e Å^−3^
Δρ_min_ = −0.15 e Å^−3^

### 

Data collection: *SMART* (Bruker, 2000 ▶); cell refinement: *SAINT* (Bruker, 2000 ▶); data reduction: *SAINT*; program(s) used to solve structure: *SHELXS97* (Sheldrick, 2008 ▶); program(s) used to refine structure: *SHELXL97* (Sheldrick, 2008 ▶); molecular graphics: *SHELXTL* (Sheldrick, 2008 ▶); software used to prepare material for publication: *SHELXTL*, *PARST* (Nardelli, 1995 ▶) and *PLATON* (Spek, 2009 ▶).

## Supplementary Material

Crystal structure: contains datablock(s) global, I. DOI: 10.1107/S1600536812042390/pv2594sup1.cif

Structure factors: contains datablock(s) I. DOI: 10.1107/S1600536812042390/pv2594Isup2.hkl

Supplementary material file. DOI: 10.1107/S1600536812042390/pv2594Isup3.cml

Additional supplementary materials:  crystallographic information; 3D view; checkCIF report

## Figures and Tables

**Table 1:** Hydrogen-bond geometry (Å, °)

*D*—H⋯*A*	*D*—H	H⋯*A*	*D*⋯*A*	*D*—H⋯*A*
N1—H1*A*⋯O2	0.91 (2)	1.90 (2)	2.608 (2)	133 (2)
O3—H3*A*⋯O1^i^	0.90 (3)	1.75 (3)	2.631 (2)	167 (2)
O4—H4*A*⋯N2	0.85 (2)	1.89 (2)	2.658 (2)	151 (2)
C8—H8*A*⋯O3^ii^	0.93	2.33	3.189 (2)	153

Symmetry codes: (i) 
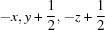
; (ii) 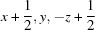.

**Fig. 1.**
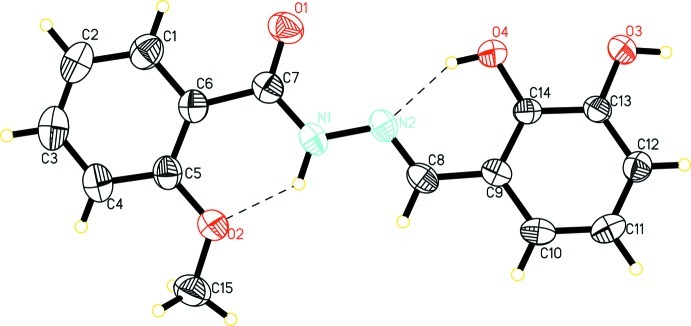
The molecular structure of the title compound showing the atom numbering scheme and N—-H···O and O—H···N intramolecular hydrogen bonds (dotted lines). Displacement ellipsoids are drawn at the 30% probability level. H atoms are presented as small spheres of arbitrary radius.

**Fig. 2.**
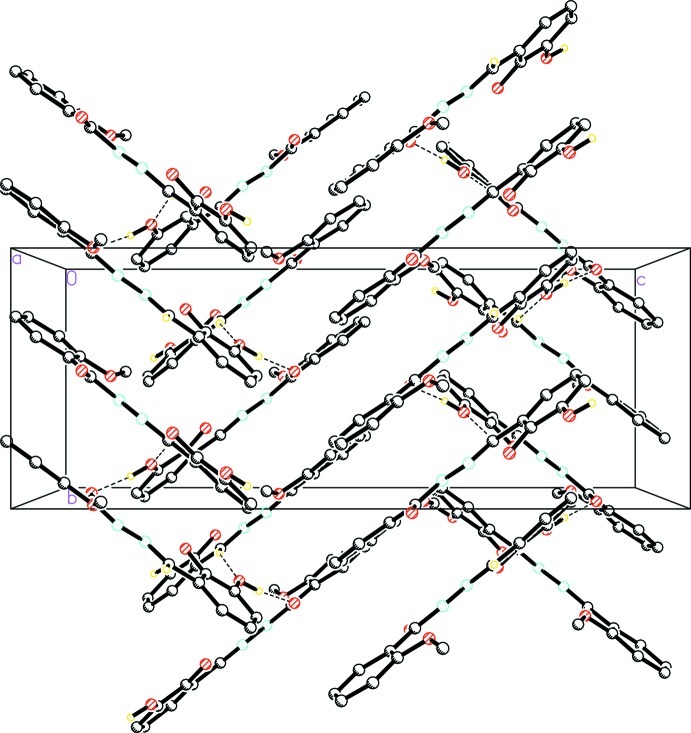
A view of the O—-H···O and C—H···O hydrogen bonds (dotted lines) in the crystal structure of the title compound. H atoms non-participating in hydrogen-bonding were omitted for clarity.

## supplementary crystallographic information

### Comment

Schiff bases represent an important group of organic compounds with a wide range of medicinal applications (Panneerselvam *et al.*, 2009; Khan *et al.*, 2009; Jarahpour *et al.*, 2007). The title Schiff base was synthesize as a part of our ongoing resaerch to study different bioactive organic compounds.

The bond lengths and angle in the title compound (Fig. 1) are similar to the corresponding bond lengths and bond angles reported in structurally realted Schiff bases (Taha *et al.*, 2012; Promdet *et al.*, 2011). The *E* configuration of azomethine oelfinic bond is stabilized by two intramolecular N1—H1A···O2, O4—H4A···N2 hydrogen bonds. O3—H3A···O1 and C8—H8A···O3 hydrogen bonds play important roles in stabilizing the crystal structure by forming a two-dimensional-network arranged parallel to the *bc* plane in a zig zag fashion (Table 2 and Fig. 2).

### Experimental

The title compound was synthesized by refluxing a mixture of 2-methoxybenzohydrazide (0.332 g, 2 mmol) and 2,3-dihydroxybenzaldehyde (0.276 g, 2 mmol) in methanol (40 ml) along with a catalytical amount of acetic acid for 3 hr. The progress of reaction was monitored by TLC. After completion of reaction, the solvent was evaporated under reduced vacuum to afford crude product which was recrystallized by dissolving in methanol at room temperature to obtain pure needles (0.458 g, 80% yield). All chemicals were purchased by sigma Aldrich Germany.

### Refinement

H atoms on methyl and phenyl C-atoms were positioned geometrically with C—H = 0.96 and 0.93 Å, respectively, and constrained to ride on their parent atoms with *U*_iso_(H) = 1.5*U*_eq_(methyl) 1.2*U*_eq_(aryl). The H atoms on the nitrogen and oxygen atoms were located from difference Fourier maps and refined isotropically. A rotating group model was applied to the methyl groups.

### Crystal data

**Table:**

C_15_H_14_N_2_O_4_	*F*(000) = 1200
*M**_r_* = 286.28	*D*_x_ = 1.376 Mg m^−^^3^
Orthorhombic, *P**b**c**a*	Mo *K*α radiation, λ = 0.71073 Å
Hall symbol: -P 2ac 2ab	Cell parameters from 1873 reflections
*a* = 14.1479 (17) Å	θ = 2.9–22.2°
*b* = 8.6567 (11) Å	µ = 0.10 mm^−^^1^
*c* = 22.570 (3) Å	*T* = 273 K
*V* = 2764.2 (6) Å^3^	Plate, colorles
*Z* = 8	0.56 × 0.18 × 0.04 mm

### Data collection

**Table:**

Bruker SMART APEX CCD area-detector diffractometer	2574 independent reflections
Radiation source: fine-focus sealed tube	1658 reflections with *I* > 2σ(*I*)
Graphite monochromator	*R*_int_ = 0.042
ω scan	θ_max_ = 25.5°, θ_min_ = 1.8°
Absorption correction: multi-scan (*SADABS*; Bruker, 2000)	*h* = −15→17
*T*_min_ = 0.945, *T*_max_ = 0.996	*k* = −10→10
15288 measured reflections	*l* = −27→26

### Refinement

**Table:**

Refinement on *F*^2^	Primary atom site location: structure-invariant direct methods
Least-squares matrix: full	Secondary atom site location: difference Fourier map
*R*[*F*^2^ > 2σ(*F*^2^)] = 0.042	Hydrogen site location: inferred from neighbouring sites
*wR*(*F*^2^) = 0.115	H atoms treated by a mixture of independent and constrained refinement
*S* = 1.03	*w* = 1/[σ^2^(*F*_o_^2^) + (0.050*P*)^2^ + 0.1101*P*] where *P* = (*F*_o_^2^ + 2*F*_c_^2^)/3
2574 reflections	(Δ/σ)_max_ < 0.001
203 parameters	Δρ_max_ = 0.14 e Å^−^^3^
0 restraints	Δρ_min_ = −0.15 e Å^−^^3^

### Special details

**Table:**

Geometry. All e.s.d.'s (except the e.s.d. in the dihedral angle between two l.s. planes) are estimated using the full covariance matrix. The cell e.s.d.'s are taken into account individually in the estimation of e.s.d.'s in distances, angles and torsion angles; correlations between e.s.d.'s in cell parameters are only used when they are defined by crystal symmetry. An approximate (isotropic) treatment of cell e.s.d.'s is used for estimating e.s.d.'s involving l.s. planes.
Refinement. Refinement of *F*^2^ against ALL reflections. The weighted *R*-factor *wR* and goodness of fit *S* are based on *F*^2^, conventional *R*-factors *R* are based on *F*, with *F* set to zero for negative *F*^2^. The threshold expression of *F*^2^ > σ(*F*^2^) is used only for calculating *R*-factors(gt) *etc*. and is not relevant to the choice of reflections for refinement. *R*-factors based on *F*^2^ are statistically about twice as large as those based on *F*, and *R*- factors based on ALL data will be even larger.

### Fractional atomic coordinates and isotropic or equivalent isotropic displacement parameters (Å^2^)

**Table:**

	*x*	*y*	*z*	*U*_iso_*/*U*_eq_	
O1	0.14939 (10)	−0.02998 (18)	0.40701 (6)	0.0774 (5)	
O2	0.43755 (10)	−0.00963 (18)	0.38297 (7)	0.0813 (5)	
O3	−0.04853 (10)	0.35880 (19)	0.18114 (7)	0.0812 (5)	
H3A	−0.0756 (18)	0.407 (3)	0.1500 (12)	0.124 (11)*	
O4	0.04345 (10)	0.21110 (17)	0.26425 (7)	0.0705 (4)	
H4A	0.0826 (17)	0.178 (3)	0.2896 (11)	0.106 (9)*	
N1	0.26779 (14)	0.08816 (19)	0.35803 (7)	0.0591 (5)	
H1A	0.3313 (16)	0.098 (3)	0.3523 (9)	0.087 (8)*	
N2	0.20663 (11)	0.16136 (18)	0.31942 (7)	0.0569 (4)	
C1	0.26911 (17)	−0.1677 (2)	0.48710 (9)	0.0752 (6)	
H1B	0.2044	−0.1659	0.4943	0.090*	
C2	0.3267 (2)	−0.2513 (3)	0.52416 (10)	0.0900 (7)	
H2B	0.3011	−0.3049	0.5560	0.108*	
C3	0.4220 (2)	−0.2550 (3)	0.51384 (11)	0.0924 (8)	
H3B	0.4611	−0.3115	0.5389	0.111*	
C4	0.46035 (17)	−0.1762 (3)	0.46702 (10)	0.0793 (7)	
H4B	0.5252	−0.1801	0.4603	0.095*	
C5	0.40271 (15)	−0.0908 (2)	0.42963 (9)	0.0628 (5)	
C6	0.30501 (15)	−0.0859 (2)	0.43918 (8)	0.0575 (5)	
C7	0.23441 (15)	−0.0071 (2)	0.40039 (8)	0.0576 (5)	
C8	0.24524 (13)	0.2483 (2)	0.28070 (8)	0.0572 (5)	
H8A	0.3106	0.2591	0.2809	0.069*	
C9	0.19093 (12)	0.3307 (2)	0.23652 (8)	0.0498 (5)	
C10	0.23670 (14)	0.4340 (2)	0.19876 (8)	0.0623 (5)	
H10A	0.3015	0.4492	0.2026	0.075*	
C11	0.18796 (15)	0.5134 (2)	0.15615 (9)	0.0661 (6)	
H11A	0.2194	0.5829	0.1317	0.079*	
C12	0.09193 (14)	0.4901 (2)	0.14946 (8)	0.0585 (5)	
H12A	0.0588	0.5437	0.1204	0.070*	
C13	0.04530 (13)	0.3880 (2)	0.18569 (8)	0.0532 (5)	
C14	0.09401 (12)	0.3093 (2)	0.23002 (7)	0.0494 (5)	
C15	0.53330 (16)	−0.0285 (3)	0.36517 (11)	0.0872 (7)	
H15A	0.5444	0.0292	0.3295	0.131*	
H15B	0.5458	−0.1359	0.3581	0.131*	
H15C	0.5743	0.0086	0.3959	0.131*	

### Atomic displacement parameters (Å^2^)

**Table:**

	*U*^11^	*U*^22^	*U*^33^	*U*^12^	*U*^13^	*U*^23^
O1	0.0578 (10)	0.1019 (12)	0.0724 (10)	−0.0083 (8)	−0.0062 (7)	−0.0100 (8)
O2	0.0576 (10)	0.0926 (11)	0.0937 (11)	0.0069 (8)	0.0005 (8)	0.0279 (9)
O3	0.0449 (9)	0.1000 (12)	0.0987 (12)	−0.0024 (7)	−0.0091 (8)	0.0351 (10)
O4	0.0515 (9)	0.0821 (10)	0.0779 (10)	−0.0071 (7)	0.0010 (7)	0.0256 (8)
N1	0.0526 (11)	0.0636 (11)	0.0611 (10)	0.0055 (9)	−0.0122 (9)	−0.0064 (9)
N2	0.0563 (10)	0.0587 (10)	0.0558 (10)	0.0057 (8)	−0.0112 (8)	−0.0083 (8)
C1	0.0884 (17)	0.0784 (15)	0.0588 (13)	−0.0058 (13)	0.0001 (12)	−0.0060 (12)
C2	0.123 (2)	0.0840 (17)	0.0635 (15)	0.0011 (17)	−0.0016 (15)	0.0123 (13)
C3	0.120 (2)	0.0834 (17)	0.0741 (16)	0.0164 (17)	−0.0178 (15)	0.0069 (14)
C4	0.0828 (17)	0.0764 (15)	0.0787 (15)	0.0075 (12)	−0.0191 (13)	0.0026 (14)
C5	0.0692 (15)	0.0594 (12)	0.0597 (12)	−0.0002 (11)	−0.0112 (11)	−0.0026 (11)
C6	0.0672 (14)	0.0543 (11)	0.0512 (12)	−0.0023 (10)	−0.0087 (10)	−0.0108 (10)
C7	0.0594 (14)	0.0605 (12)	0.0528 (12)	−0.0033 (10)	−0.0059 (10)	−0.0153 (10)
C8	0.0467 (11)	0.0611 (11)	0.0638 (12)	−0.0008 (10)	−0.0078 (10)	−0.0143 (11)
C9	0.0457 (11)	0.0503 (10)	0.0533 (11)	−0.0015 (8)	0.0004 (8)	−0.0113 (9)
C10	0.0489 (12)	0.0679 (13)	0.0700 (13)	−0.0115 (10)	0.0054 (10)	−0.0082 (11)
C11	0.0723 (14)	0.0613 (13)	0.0648 (13)	−0.0153 (11)	0.0126 (11)	0.0012 (11)
C12	0.0644 (13)	0.0547 (11)	0.0565 (11)	0.0022 (10)	0.0021 (10)	0.0014 (10)
C13	0.0439 (11)	0.0541 (11)	0.0616 (12)	0.0017 (9)	0.0016 (9)	0.0011 (10)
C14	0.0465 (11)	0.0487 (10)	0.0531 (11)	−0.0023 (8)	0.0063 (9)	−0.0007 (9)
C15	0.0601 (15)	0.0902 (17)	0.1112 (19)	0.0038 (12)	0.0052 (13)	0.0059 (15)

### Geometric parameters (Å, º)

**Table:**

O1—C7	1.228 (2)	C4—C5	1.387 (3)
O2—C5	1.359 (2)	C4—H4B	0.9300
O2—C15	1.422 (2)	C5—C6	1.400 (3)
O3—C13	1.355 (2)	C6—C7	1.493 (3)
O3—H3A	0.90 (3)	C8—C9	1.447 (2)
O4—C14	1.353 (2)	C8—H8A	0.9300
O4—H4A	0.85 (2)	C9—C14	1.392 (2)
N1—C7	1.348 (2)	C9—C10	1.395 (2)
N1—N2	1.382 (2)	C10—C11	1.368 (3)
N1—H1A	0.91 (2)	C10—H10A	0.9300
N2—C8	1.276 (2)	C11—C12	1.382 (3)
C1—C2	1.373 (3)	C11—H11A	0.9300
C1—C6	1.389 (3)	C12—C13	1.373 (2)
C1—H1B	0.9300	C12—H12A	0.9300
C2—C3	1.368 (3)	C13—C14	1.393 (2)
C2—H2B	0.9300	C15—H15A	0.9600
C3—C4	1.370 (3)	C15—H15B	0.9600
C3—H3B	0.9300	C15—H15C	0.9600
			
C5—O2—C15	120.34 (17)	N2—C8—C9	122.37 (17)
C13—O3—H3A	113.0 (17)	N2—C8—H8A	118.8
C14—O4—H4A	104.7 (17)	C9—C8—H8A	118.8
C7—N1—N2	120.55 (18)	C14—C9—C10	118.61 (17)
C7—N1—H1A	120.2 (14)	C14—C9—C8	122.01 (16)
N2—N1—H1A	119.1 (14)	C10—C9—C8	119.38 (17)
C8—N2—N1	115.74 (17)	C11—C10—C9	121.16 (19)
C2—C1—C6	121.7 (2)	C11—C10—H10A	119.4
C2—C1—H1B	119.1	C9—C10—H10A	119.4
C6—C1—H1B	119.1	C10—C11—C12	119.95 (18)
C3—C2—C1	119.5 (2)	C10—C11—H11A	120.0
C3—C2—H2B	120.2	C12—C11—H11A	120.0
C1—C2—H2B	120.2	C13—C12—C11	120.08 (18)
C2—C3—C4	120.7 (2)	C13—C12—H12A	120.0
C2—C3—H3B	119.7	C11—C12—H12A	120.0
C4—C3—H3B	119.7	O3—C13—C12	123.07 (17)
C3—C4—C5	120.1 (2)	O3—C13—C14	116.58 (17)
C3—C4—H4B	119.9	C12—C13—C14	120.34 (17)
C5—C4—H4B	119.9	O4—C14—C9	123.01 (16)
O2—C5—C4	122.3 (2)	O4—C14—C13	117.13 (16)
O2—C5—C6	117.50 (17)	C9—C14—C13	119.84 (16)
C4—C5—C6	120.2 (2)	O2—C15—H15A	109.5
C1—C6—C5	117.72 (19)	O2—C15—H15B	109.5
C1—C6—C7	116.4 (2)	H15A—C15—H15B	109.5
C5—C6—C7	125.77 (19)	O2—C15—H15C	109.5
O1—C7—N1	121.88 (19)	H15A—C15—H15C	109.5
O1—C7—C6	120.7 (2)	H15B—C15—H15C	109.5
N1—C7—C6	117.45 (19)		
			
C7—N1—N2—C8	−179.58 (16)	C5—C6—C7—N1	−11.4 (3)
C6—C1—C2—C3	−0.2 (3)	N1—N2—C8—C9	178.90 (14)
C1—C2—C3—C4	0.0 (4)	N2—C8—C9—C14	−6.0 (3)
C2—C3—C4—C5	0.4 (4)	N2—C8—C9—C10	174.56 (16)
C15—O2—C5—C4	9.8 (3)	C14—C9—C10—C11	0.1 (3)
C15—O2—C5—C6	−170.20 (18)	C8—C9—C10—C11	179.55 (16)
C3—C4—C5—O2	179.4 (2)	C9—C10—C11—C12	−0.9 (3)
C3—C4—C5—C6	−0.6 (3)	C10—C11—C12—C13	0.3 (3)
C2—C1—C6—C5	−0.1 (3)	C11—C12—C13—O3	−179.38 (18)
C2—C1—C6—C7	176.70 (19)	C11—C12—C13—C14	1.1 (3)
O2—C5—C6—C1	−179.52 (17)	C10—C9—C14—O4	179.63 (16)
C4—C5—C6—C1	0.5 (3)	C8—C9—C14—O4	0.2 (3)
O2—C5—C6—C7	4.1 (3)	C10—C9—C14—C13	1.2 (2)
C4—C5—C6—C7	−175.94 (17)	C8—C9—C14—C13	−178.16 (16)
N2—N1—C7—O1	−1.4 (3)	O3—C13—C14—O4	0.1 (2)
N2—N1—C7—C6	178.09 (14)	C12—C13—C14—O4	179.65 (16)
C1—C6—C7—O1	−8.4 (3)	O3—C13—C14—C9	178.57 (16)
C5—C6—C7—O1	168.06 (18)	C12—C13—C14—C9	−1.9 (3)
C1—C6—C7—N1	172.13 (16)		

### Hydrogen-bond geometry (Å, º)

**Table:**

*D*—H···*A*	*D*—H	H···*A*	*D*···*A*	*D*—H···*A*
N1—H1*A*···O2	0.91 (2)	1.90 (2)	2.608 (2)	133 (2)
O3—H3*A*···O1^i^	0.90 (3)	1.75 (3)	2.631 (2)	167 (2)
O4—H4*A*···N2	0.85 (2)	1.89 (2)	2.658 (2)	151 (2)
C8—H8*A*···O3^ii^	0.93	2.33	3.189 (2)	153

Symmetry codes: (i) −*x*, *y*+1/2, −*z*+1/2; (ii) *x*+1/2, *y*, −*z*+1/2.
